# Abrin Toxicity and Bioavailability after Temperature and pH Treatment

**DOI:** 10.3390/toxins9100320

**Published:** 2017-10-13

**Authors:** Christina C. Tam, Thomas D. Henderson, Larry H. Stanker, Xiaohua He, Luisa W. Cheng

**Affiliations:** Foodborne Toxin Detection and Prevention Research Unit, Western Regional Research Center, Agricultural Research Services, United States Department of Agriculture, 800 Buchanan Street, Albany, CA 94710, USA; christina.tam@ars.usda.gov (C.C.T.); thomas.henderson@ars.usda.gov (T.D.H.II); larry.stanker@ars.usda.gov (L.H.S.); Xiaohua.he@ars.usda.gov (X.H.)

**Keywords:** abrin, *Abrus precatorius*, mouse bioassay, food safety, temperature stability, pH stability

## Abstract

Abrin, one of most potent toxins known to man, is derived from the rosary pea (jequirity pea), *Abrus precatorius* and is a potential bioterror weapon. The temperature and pH stability of abrin was evaluated with an in vitro cell free translation (CFT) assay, a Vero cell culture cytotoxicity assay, and an in vivo mouse bioassay. pH treatment of abrin had no detrimental effect on its stability and toxicity as seen either in vitro or in vivo. Abrin exposure to increasing temperatures did not completely abrogate protein translation. In both the cell culture cytotoxicity model and the mouse bioassay, abrin’s toxic effects were completely abrogated if the toxin was exposed to temperatures of 74 °C or higher. In the cell culture model, 63 °C-treated abrin had a 30% reduction in cytotoxicity which was validated in the in vivo mouse bioassay with all mice dying but with a slight time-to-death delay as compared to the non-treated abrin control. Since temperature inactivation did not affect abrin’s ability to inhibit protein synthesis (A-chain), we hypothesize that high temperature treatment affected abrin’s ability to bind to cellular receptors (affecting B-chain). Our results confirm the absolute need to validate in vitro cytotoxicity assays with in vivo mouse bioassays.

## 1. Introduction

*Abrus precatorius*, native to India as well as tropical/subtropical regions, was originally introduced to Florida and the Caribbean [1,2] but has established itself in many parts of the southeastern United States [3]. The seeds of *A. precatorius*, also known as jequirity, rosary pea, and Crab’s Eye, contain one of the most potent toxins in the world, abrin. These seeds have traditionally been made into bracelets and necklaces for use as jewelry as well as rosary beads.

Abrin and ricin are both members of the Type II family of ribosome-inactivating proteins (RIP) that inhibit eukaryotic protein synthesis leading to apoptosis and cell death. Abrin consists of an A-chain of ≈30 kDa and a B-chain of ≈33 kDa linked together by a single disulfide bond. Abrin A-chain is an N-glycosidase that cleaves the C–N bond of adenine at position 4324 on the 28S ribosomal RNA thereby preventing ribosomes from binding to elongation factor (EF) 1 and 2, leading to inhibition of protein synthesis and eventual cell death [2,3,4,5,6]. Abrin B-chain is a lectin that binds to cell surface carbohydrate receptors that facilitate receptor-mediated endocytosis of the A-B toxin. After internalization and reduction of the disulfide bond, the A-chain interacts with ribosomes in the cytosol thereby causing the inhibition of protein synthesis.

Abrin isolated from *Abrus precatorius* seeds has many different potential isoforms [7,8,9]. Three to four isoforms have been isolated by different laboratories and reported to have different toxin activities or median lethal doses (LD_50_) [7,9,10,11,12,13,14]. Our abrin toxin preparations also contained a 120 kDa heterotetrameric species, agglutinin (APA-1). APA-1 consists of two A-chains and two B-chains that are stabilized through hydrophilic and hydrophobic interactions [15]. APA-1 has reduced toxicity as compared to abrin [7,16,17].

Abrin intoxication can occur via the gastrointestinal, inhalational, or cutaneous routes. Accidental abrin intoxication cases have been primarily attributed to children having ingested *A. precatorius* seeds, usually with no serious effects if the integrity of the seed was not compromised [15]. Since there is no antidote for abrin poisoning, the only treatment available is supportive care to mitigate the effects of the toxin. In the case of abrin intoxication via ingestion, the current treatment consists of induction of emesis, gastric lavage, activated charcoal, and whole bowel irrigation [3].

The LD_50_ of abrin for humans has been reported to be from 10 to 1000 μg/kg via oral ingestion and 3.3 μg/kg if injected [18]. In mice, abrin has been shown to be 31.4 times more lethal than ricin at 0.7 μg/kg vs. 22 μg/kg when given intravenously [19]. Since abrin is so much more toxic than ricin with no remedy for intoxication, one can imagine the devastation that would ensue if our nation’s food supply were to be contaminated deliberatively in a terrorist attack. To help mitigate this potential disaster, we would need to evaluate the effectiveness of the current food safety processing practices in inactivating abrin toxicity.

Previous work on the thermal and pH stability of protein contaminants such as abrin have primarily used calorimetry, intrinsic fluorescence, ELISA, and cell culture cytotoxicity assays to measure the activity of abrin [20,21,22]. The results of these studies suggest that abrin is extremely heat stable and pH tolerant, and that food matrices such as dairy negatively affect the biological activity of the toxin [22]. However, these studies do not assess the actual in vivo impact of these inactivation strategies in a mouse bioassay to mimic a true intoxication scenario. In our current study, we will determine the thermal and pH stability of abrin via three different assays: (1) an in vitro cell free translation assay to measure active abrin A-chain activity; (2) an in vitro Vero cell cytotoxicity assay; and (3) a validation of the in vitro results with a mouse bioassay which will mimic a real-life intoxication model and evaluate the ability of the toxin to be absorbed and cause cytotoxicity.

## 2. Results

### 2.1. Abrin Toxin is Heterogeneous

Work in many laboratories has established that the isolation of abrin from *Abrus precatorius* seeds can yield three to four isoforms [7,8,9]. These isoforms can vary slightly at the amino acid level and have different toxin activity levels. Interestingly, these seed preparations also contain a related 120 kDa heterotetrameric species, agglutinin (APA-1). APA-1 consists of two A-chains and two B-chains that are stabilized through hydrophilic and hydrophobic interactions [15]. Agglutinin was shown to have reduced toxicity as compared to abrin [7,16,17].

**Figure 1:**
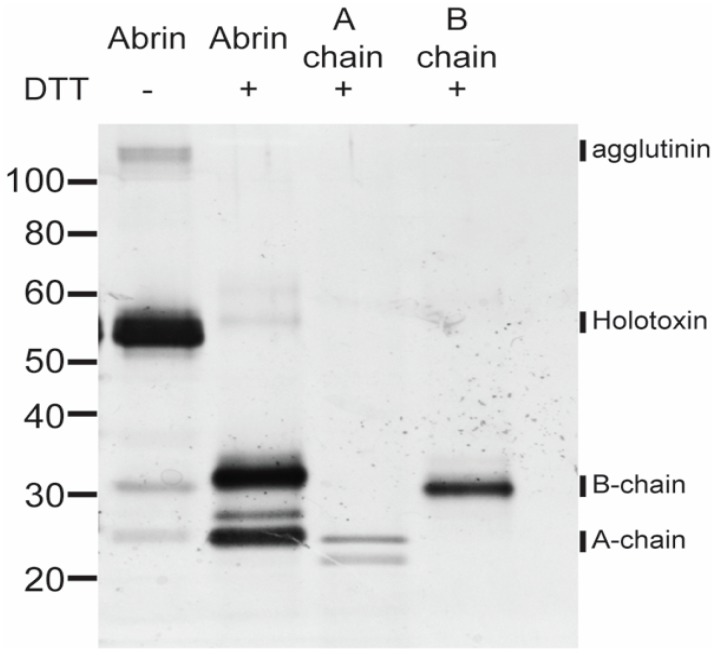
SDS-PAGE analysis of heterogeneous toxin complexes and subunits. An amount of 100 ng per lane of sample (abrin, abrin A-chain, abrin B-chain) treated or not treated with 0.05 M DTT was loaded onto a NuPAGE 4–12% Bis-Tris gel and subjected to SDS-PAGE electrophoresis. The gel was silver stained with the SilverXpress kit. In the absence of the reducing agent, abrin predominantly consists of the holotoxin with small amounts of agglutinin, A-chain, and B-chain. Once reduced with DTT, abrin is almost all reduced to the smaller individual A- and B-chains. The abrin A-chain control sample with DTT has two predominant A-chain species. The reduced abrin B-chain control has one predominant species that is of higher molecular weight than the individual A-chain control.

Due to these reports, we sought to determine the components of our “abrin” toxin stock before further experiments in order to characterize its stability and bioavailability. Abrin with or without the reducing agent, DTT, along with the individual abrin A-and B-chain controls were loaded at a concentration of 100 ng per well and separated by SDS-PAGE electrophoresis. This gel was subsequently silver-stained and the components in each lane were analyzed. As seen in Figure 1, abrin without DTT had one predominant molecular weight species ≈ 55–60 kDa (holotoxin). A higher molecular weight species ≈ 120 kDa (agglutinin) and two smaller molecular weight species ≈ 25–35 kDa (individual B- and A-chains) were also present in this extract, but in much smaller amounts than the abrin holotoxin. In the presence of DTT, abrin is mostly reduced to the individual A- and B-chains. The trace amounts of the two higher molecular weight species found in this sample could be from residual non-reduced abrin holotoxin and/or agglutinin which was not fully reduced to individual A- and B-chains. The abrin A-chains seem to have at least two different molecular weight species, indicating different A-chain isomers. The B-chain control has one predominant band ≈ 30–35 kDa whereas the reduced abrin sample indicates heterogeneity of B-chains.

### 2.2. Cell Free Translation Assay Measurements of Temperature Effects on Abrin Stability

Abrin has been reported to be very stable and able to withstand extreme heat and cold [15,20,21,22]. Similarly, other RIPs such as the Type I RIP saporin have been reported to be highly stable [23,24]. Since most known abrin poisonings are accidental, studies to understand how abrin is affected by food processing protocols in the case of deliberate poisoning is of critical importance. Work on other plant lectins have shown that they are very heat-stable and are able to withstand denaturation at 80 °C [25]. The food industry treats foods at certain temperatures as a way to inactivate pathogens and toxins. We wondered if these same temperature inactivation protocols would have any applicable effects in inactivating abrin.

**Figure 2:**
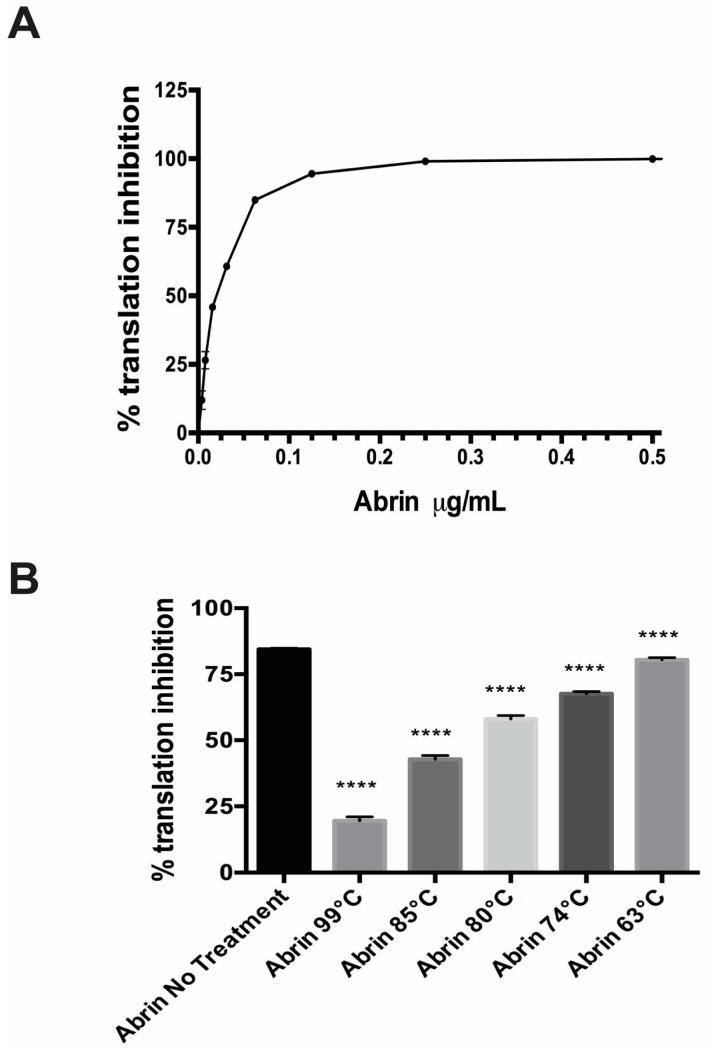
Translation inhibition by the plant toxin Abrin was abrogated by exposure to high temperature. (**A**) A representative standard curve shows increasing translation inhibition until saturation as one increased the abrin concentration in the in vitro cell free translation (CFT) assay run in parallel with the experimental samples shown in (**B**). Values represent means of triplicate samples ± SD. (**B**) Cell free translation assay using a single concentration of toxin (100 ng/mL) for all the different conditions. Increasing the temperature that abrin is exposed to decreases the translational inhibition seen in the CFT assay. Values represent means of triplicate samples ± SD. Statistical significance was determined by two-tailed unpaired Student’s *t*-test, (****) *p* < 0.0001. Two independent experiments in triplicate were performed and one representative data set is presented.

To assay for active toxin, we took advantage of the fact that abrin, like ricin, are both plant toxins that are ribosome-inactivating proteins. We modified an established cell free translation (CFT) assay used for both ricin and Shiga toxin (Stx2) [26,27] to detect active abrin. In Figure 2A, a representative abrin toxin standard curve shows that increasing concentrations of toxin correlate with increasing translation inhibition (decreasing ability to translate the reporter luciferase mRNA, therefore less luminescence measured). At concentrations above 125 ng/mL of toxin, the CFT assay is unable to respond to changes in toxin concentration since translation is completely inhibited. Therefore, we decided to validate all in vitro toxin cell free translation activity assays using a single concentration of 100 ng/mL of toxin because this dose shows high translation inhibition but allows for any detrimental effects on toxin function to be noticed.

The abrin toxin was exposed to increasing temperatures (63 °C, 74 °C, 80 °C, 85 °C, 99 °C) for 3 min in a thermocycler. Since food manufacturing processes vary tremendously depending on the actual food product and the pathogen and/or toxin chosen as the target to be inactivated, these temperatures and times were chosen based upon previous work in the laboratory for ricin (unpublished) as well as guidelines by the U.S. Food & Drug Administration to inactivate 90% of the pathogens and toxins present in sample [28,29,30]. These conditions certainly may not reflect the actual food processing conditions. The CFT assay shows that abrin is thermally stable since exposure to the highest temperature of 99 °C for 3 min did not completely abrogate its ability to inhibit luciferase mRNA translation. Samples treated at 99 °C had ~19.55 ± 0.61% translation inhibition while abrin without treatment was at ~84.5 ± 0.2% (*p* < 0.0001). When abrin was exposed to 85 °C for 3 min, toxin inhibition of translation activity decreased ≈ 50% to ~ 42.8 ± 0.6% (*p* < 0.0001) as compared to the untreated toxin control. Exposure of abrin to 63 °C for 3 min, though statistically significant, had the least impact in the CFT assay at ~80.43 ± 0.36% as compared to abrin with no treatment (*p* < 0.0001).

### 2.3. Cytotoxic Effects of Temperature-Treated Abrin on Vero Cells

Since abrin showed a temperature-dependent decrease in translation inhibition in the cell free translation assay, we decided to evaluate the cytotoxic effect of the toxins in the well-known Vero (African green monkey epithelial) cell culture model that has been used extensively in toxin research [31,32]. In the Vero cell assay, the cytotoxic effects of abrin were completely abrogated if the toxin was treated with temperatures ≥ 74 °C for 3 min, similar to the cells with Dulbeco’s Modified Eagle Medium (DMEM) alone (Table 1, *p* < 0.0001). These results were completely different from the results shown in Figure 2B with the CFT, which measures the catalytic function of the toxin A-chain. The 63 °C-treated abrin, though still toxic to cells, had a statistically significant ≈ 30% reduction in cytotoxicity compared with the untreated abrin in the cell culture assay, but showed a decrease of only 4% of catalytic abrin A-chain activity in the translation inhibition CFT assay (Figure 2B).

### 2.4. Mouse Bioassay Determination of Temperature Effects on Abrin Stability and Toxicity

**Figure 3:**
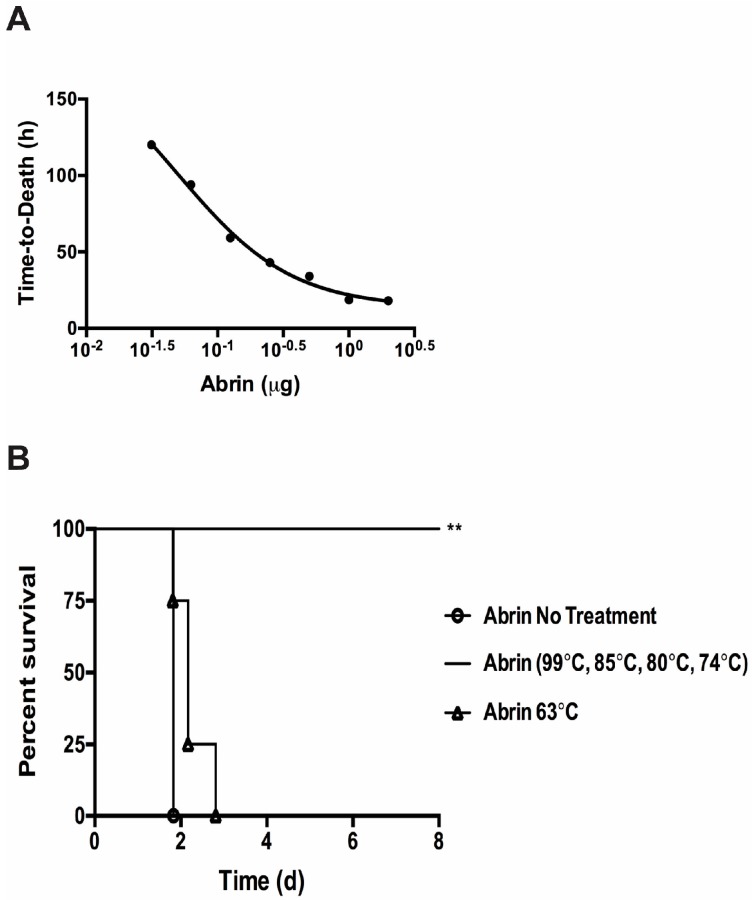
Temperature effects on abrin toxicity in the intravenous mouse bioassay. (**A**) A series of known abrin samples were injected into mice tail vein injection (iv) to derive time-to-death standard curves and LD_50_ values. Data was combined from two independent experiments from seven dosage levels consisting of a total of *n* = 6 to 8 mice per dose. The data was plotted using the log (inhibitor) vs. response (three parameter) curve on GraphPad Prism 6. R^2^ = 0.8257; (**B**) Temperature treated toxins were administered to mice iv at a lethal dose of 1 μg per mouse (*n* = 4 mice per experimental condition). Mice given abrin treated at 74 °C or higher all survived as compared to the untreated controls (**, *p* = 0.0082). The time-to-death delay seen from mice given the abrin treated at 63 °C was not statistically significant (*p* = 0.1580). Two independent experiments were performed and one representative set of survival curves is shown. Survival curves were plotted for each condition and the log-rank (Mantel–Cox) test was used to evaluate statistical significance on GraphPad Prism 6.

The Vero cell cytotoxicity results suggest that substantial amounts of abrin remain active after thermal exposure at ≤ 63 °C (Table 1). We wanted to validate these cell culture cytotoxicity results with the in vivo mouse bioassay. From the Vero cell assay, one can expect intoxication and death in the mouse bioassay for abrin treated at ≤ 63 °C. Since the mean lethal dose (LD_50_) varies widely due to the route of intoxication as well as the activity of the toxin extracts, one must establish an LD_50_ and mouse bioassay for individual toxin batch preparations. The mouse tail vein injection (iv) model was used to assess the toxicity of abrin as well as temperature exposed toxin solutions. Groups of mice were injected iv with seven different dosages of abrin starting with the highest concentration at 2 μg/mouse decreasing two-fold in phosphate buffer + 0.2% gelatin (Figure 3A). Mice were monitored for at least 8 days and standard time-to-death (median survival) vs. toxin dose curve was plotted. The time-to-death curve plotted for abrin is a log (inhibitor) vs. response (three parameter) curve rather than a linear regression model (R^2^ = 0.8257). Intoxication with doses above 0.25 μg/mouse caused the rapid death of all members of the group within the first 48 h. Mice injected with abrin at 0.125 μg/mouse almost all succumbed to death by 72 h whereas lower doses did not cause death and/or show intoxication. An average LD_50_ value of 3.3 μg/kg for our abrin stock using the iv route of infection was derived from the LD_50_ values calculated using the Weil method and the Reed and Muench method [33,34]. This value is higher than the 0.7 μg/kg iv reported in the literature [35]. An earlier study using an abrin toxin stock from the same supplier has determined a LD_50_ value of > 1 mg/kg for oral intoxication and 2–20 μg/kg for iv treated mice [15]. Our LD_50_ for the iv infection model of 3.3 μg/kg is similar to that of the previous study.

To determine the effectiveness of abrin inactivation by thermal treatment in vivo, treated and non-treated toxins were injected iv into groups of mice (*n* = 4 per experimental condition) at 1 μg/mouse (equal to 13 x mouse iv LD_50_). Survival curves were calculated and the results are shown in Figure 3B. Mice that were given abrin inactivated at 74 °C or higher survived compared to the abrin no treatment control (abrin no treatment median survival ≈ 1.83 days vs. abrin ≥ 74 °C median survival ≈ end of experiment, log-rank (Mantel–Cox) *p* = 0.0082) in agreement with the Vero cell cytotoxicity assay (Table 1). Mice given abrin treated at 63 °C all died similarly to the abrin no heat treatment control but with a slight time-to-death delay that was not statistically significant (abrin no treatment median survival was ≈ 1.83 days vs. abrin 63 °C median survival at ≈ 2.167 days, log-rank (Mantel–Cox) *p* = 0.1580). Abrin treated at 63 °C similarly was still able to cause a significant amount of cytotoxicity in the Vero cell cytotoxicity assay but was reduced in comparison to the non-treated abrin control (Table 1). This reduction in cytotoxicity is reflected in the slight time-to-death delay seen in the in vivo mouse bioassay.

### 2.5. pH Treatment of Abrin Has No Detrimental Effect on Its Function

**Figure 4:**
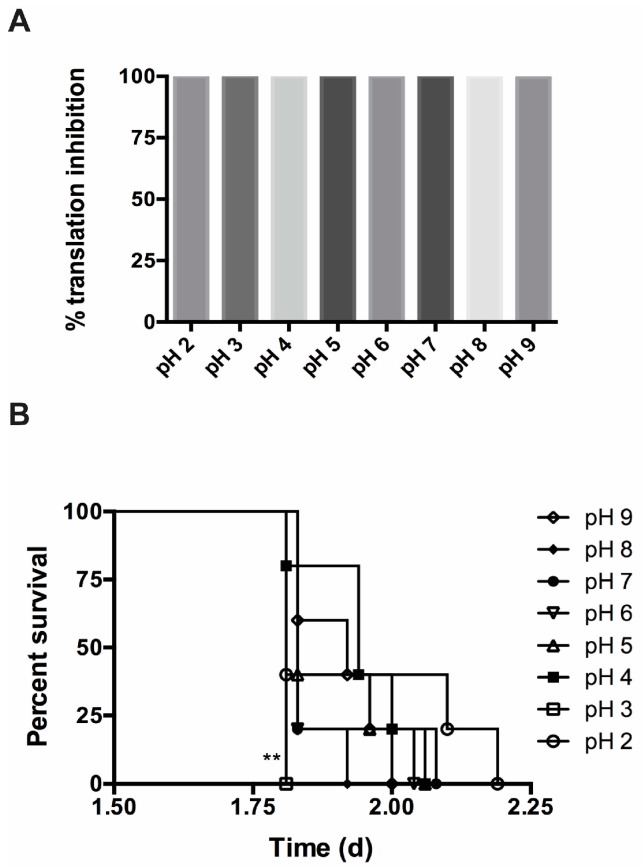
pH treatment of abrin has no detrimental effect on toxin activity. (**A**) A single concentration of 100 ng/mL abrin was used in cell free translation assays for all pH conditions. There is no significant difference between the various pH-exposed toxins. Values represent means of triplicate samples ± SD. Statistical significance was determined by two-tailed unpaired Student’s *t*-test. The data represents a single experiment; (**B**) pH-treated toxins were administered to mice iv at a lethal dose of 1 μg per mouse (*n* = 5 mice per experimental condition) for one experiment. There was no detrimental effect on abrin’s ability to cause intoxication and subsequent death. Survival curves were plotted for mice for each condition and the log-rank (Mantel–Cox) test was used to evaluate statistical significance on GraphPad Prism 6. The only statistical significant decrease in toxicity was seen in abrin treated at pH 3.0 which shortened the time-to-death compared to abrin treated at pH 7.0 (** *p* = 0.0027).

One of the main routes for abrin intoxication is via the gastrointestinal route. After oral ingestion of the toxin, abrin must travel through the digestive system, encountering a steadily decreasing pH gradient. How the toxin survives this change to an increasing acidic milieu and what effects this may have on toxin function are of critical importance. In addition, different food matrices may present a range of pH conditions that may affect toxin function. To determine the effect of pH on toxicity, abrin was subjected to different pH levels in the phosphate buffer gelatin matrix (pH 2.0–pH 9.0) for 1 h at room temperature. Gelatin was used because of the tendency of low amounts of abrin to bind to tube walls. After exposure, the toxin mixes were neutralized with phosphate buffer gelatin to pH 7.0 before addition to the CFT assay, Vero cell cytotoxicity assay, or mouse bioassay. In Figure 4A, no decrease in toxicity was observed between abrin pH 7.0 or any of the other pH samples in the cell free translation assay. pH-treated abrin was still able to inhibit luciferase mRNA translation (% translation inhibition ≅ 99.6–99.9%). Additionally, pH-treated toxin did not have significant deficient cytotoxic effects on Vero cells (Table S1). For the mouse bioassay, mice in all the experimental groups died rapidly within 2.25 days (*n* = 5 mice per experimental condition). The median survival for the various pH-treated abrin were pH 9.0 ≈ 1.92 days, pH 5.0–9.0 ≈ 1.83 days, pH 4.0 ≈ 1.94 days, and pH 2.0 ≈ 1.81 days but they were not statistically significant compared to pH 7.0-treated abrin. However, there was a statistical difference between abrin pH 7.0 and abrin pH 3.0 (abrin pH 7.0 median survival ≈ 1.83 days vs. abrin pH 3.0 median survival ≈ 1.81, log-rank (Mantel–Cox) *p* = 0.0027).

## 3. Discussion

In this study, we wanted to determine the most effective parameters to use against abrin should bioterror attacks occur on our food supply. We proceeded to evaluate the effectiveness of thermal inactivation against abrin. To measure the successful inactivation of abrin toxin activity, we utilized three different assays: two in vitro and one in vivo. As shown in Figure 2B, the in vitro cell free translation assay showed that there was a correlation between increasing temperature exposure and decreasing toxin activity. However, treatment, even at the highest temperature of 99 °C for 3 min, still gave a statistically significant translation inhibition activity of ≈20% which was approximately a 4-fold decrease from toxin that was not exposed to any thermal inactivation conditions. The heat-stability of abrin in this study is similar to previous published data that suggest that batch pasteurization at 85 °C for 30 min is required to completely inactivate ricin and abrin activity in milk when measured by ELISA [22]. When these toxins were tested for toxicity in the Vero cell model, high temperature inactivation (74 °C or higher) completely abrogated cell toxicity while 70% of the Vero cells were killed after intoxication with 63 °C-treated toxin (Table 1). The in vivo mouse bioassay validated the defect in Vero cell cytotoxicity treated with abrin exposed to ≥74 °C as well as the slight decrease in toxicity (i.e., slight time-to-death delay) with the 63 °C-treated abrin (Table 1, Figure 4B). Since we know that the in vitro CFT assay measures the catalytic ability of the A-chain to bind to ribosomes to inhibit protein synthesis, and that the cell culture cytotoxicity and mouse bioassay models account for receptor binding and enzymatic activity, these in vivo results suggest that higher temperatures did not affect A-chain catalytic activity. The decrease in toxicity thus could be due to the disruption of the B-chain’s ability to bind to the proper cell surface receptors to cause internalization of the toxin and/or cause the toxin complex to be more susceptible to degradation in vivo. To determine if proteolysis/degradation of the holotoxin or individual subunits was causing the decrease in toxicity, the toxins were examined via silver staining in the absence or presence of a reducing agent. In Figure S1, we show a similar protein profile present in the extracts after thermal or pH treatment within each silver stained gel. This indicates that there is still a significant amount of toxin left to cause cytotoxicity. Therefore, protein degradation does not seem to be the cause of the toxicity differences (Table 1, Figure 3B and Figure 4B). Future work is needed to validate our hypothesis that the thermal effect that we see of 63 °C-treated abrin is potentially due to abrin conformational changes that could affect their binding to carbohydrate receptors using antibodies that specifically recognize native lectin chains [36] and/or binding measurements to the receptors themselves.

In addition to evaluating the thermal stability of abrin, we decided to investigate the stability of the toxin at different pH. Abrin can be found in foods of different pH and toxin ingestion requires passage through increasing acidic pH in the digestive system. In addition, low pH is needed for A-chain translocation to the cytosol to bind to and affect ribosomes. Abrin toxin activity investigated from pH 2.0 to pH 9.0. pH treatment did not significantly affect the ability of abrin to inhibit the translation seen in the CFT assay (Figure 4A) or the Vero cell cytotoxicity (Table S1), nor did it inhibit its ability to cause death in the mouse bioassay (Figure 4B).

Our research reaffirms the need for in vivo mouse bioassays to validate in vitro experimental assays, as seen in the thermal stability study. Our study on abrin thermal stability suggests that the food safety procedures utilizing thermal inactivation of pathogens and toxins must be set to ≥74 °C to be truly effective. Any food safety procedures that comprise a pH only treatment may not be sufficient for abrin inactivation. Since we treated the toxin for 1 h at room temperature at the different pHs and then neutralized the solution, one must wonder if there may be differences if one increases the incubation time and/or does not neutralize the pH. Additionally, our studies were performed using a phosphate buffer gelatin and not any food matrices. Future studies should take this into account to test the effectiveness of these procedures in different food matrices, i.e., milk, beef, and eggs.

## 4. Materials and Methods

### 4.1. Materials

Abrin toxin (mixed isomers, Cat# ABR-1) was obtained from Toxin Technology, Inc. (Sarasota, FL, USA) as 1 mg lyophilized powder before resuspension with 1 mL of 1× phosphate buffered saline (PBS) pH 7.2 to give a 1 mg/mL stock stored at 4 °C. Abrin A-chain (BEI Resources, NR-43945), Abrin B-chain (BEI Resources, NR-43946) were supplied as 1 mg/mL stock stored at −20 °C. The following components for the cell free translation assay were bought from Promega (Madison, WI, USA): nuclease-treated rabbit reticulocyte lysate (L4960), complete amino acid mixture (1 mM, N2111), nuclease-free water (P1193), luciferase mRNA (1 mg/mL, L4561), and the Bright-Glo Luciferase Assay System (10 mL, E2610). The NuPAGE 4–12% Bis-Tris gels and SilverXpress kit (LC6100) were supplied from Invitrogen (Carlsbad, CA, USA).

### 4.2. Temperature Stability Assays

Abrin was diluted in 1× PBS pH 7.2 + 0.2% phosphate buffer gelatin to 10 μg/mL. Aliquots of these toxins were treated at various temperatures (63 °C, 74 °C, 80 °C, 85 °C, and 99 °C) for 3 min in a thermocycler. The toxin samples per condition were split into two for further analysis. One half of the samples were used for the cell free translation assay at a single concentration of 100 ng/mL. Statistical significance was calculated using the two-tailed unpaired Student’s *t*-test between the non-heat-treated abrin sample with each of the other heat-treated toxin samples. The other half of the heat-treated and non-heat-treated toxin samples were administered to mice iv at a lethal dose of 1 μg per mouse (*n* = 4 mice per experimental condition). These conditions were tested in two independent experiments for a total of *n* = 8 mice per experimental condition. Survival curves were generated for each condition and compared for statistical significance using GraphPad Prism 6 for each independent experiment. The log-rank (Mantel–Cox) test was used to determine statistical significance between the survival curves with *p*-values < 0.05 considered significant.

### 4.3. pH Stability Assays

Abrin was diluted to 20 μg/mL in 1× PBS + 0.2% phosphate buffer gelatin at different pH (pH 2.0–pH 9.0) for 1 h at room temperature. After this incubation, the pH-treated toxin samples were neutralized with 1× PBS + 0.2% phosphate buffer gelatin 1:1 (*v*/*v*) to generate a stock of 10 μg/mL at pH 7.0 before further analysis. One half of the samples was used for the cell free translation assay at a single concentration of 100 ng/mL. Statistical significance was calculated using the two-tailed unpaired Student’s *t*-test between the pH 7.0-treated abrin sample with each of the other different pH-treated toxin samples. The rest of the toxin samples were administered to mice iv at a lethal dose of 1 μg per mouse (*n* = 5 mice per experimental condition) in one experiment. Survival curves were generated for each condition and compared for statistical significance using GraphPad Prism 6 for each independent experiment. The log-rank (Mantel–Cox) test was used to determine statistical significance between the survival curves with *p*-values < 0.05 considered significant.

### 4.4. SDS-PAGE Electrophoresis for Silver Stain

Abrin, abrin A-chain, and abrin B-chain at the concentration of 100 ng per well in the presence or absence of a reducing agent (0.05 M DTT) were separated by sodium dodecyl sulfate-polyacrylamide gel electrophoresis with NuPAGE 4–12% Bis-Tris gels (Invitrogen) followed by silver staining using the SilverXpress kit according to the manufacturer’s instructions (Invitrogen). The silver stained gel image was acquired using the ChemiDoc MP imaging system (BIO-RAD, Hercules, CA, USA). Molecular weight standards were purchased from Invitrogen. Abrin untreated or treated with either increasing temperatures or pH in 1× PBS with 0.2% phosphate buffer gelatin were loaded at 100 ng per well as above. Electrophoresis and silver-staining procedures were followed as stated.

### 4.5. Abrin In Vitro Cell Free Translation Assay

The in vitro cell free translation assay for abrin was modified from the protocols used to detect ricin and Shiga toxin (Stx2) activity in regards to the concentration of toxins evaluated [26,27]. Briefly, serial dilutions of abrin (for standard curve) or a single concentration from 100 ng/mL (for heat-treated or pH-treated toxin experiments) were added to the translational lysate mixture: nuclease-treated rabbit reticulocyte lysate, complete amino acid mixture, RNasin, nuclease-free water, and luciferase mRNA at ratio of (*v*/*v*) [35:1:1:36:2]. The ratio of toxin to translational lysate mixture was 1:5. Fifteen microliters of lysate mixture was added to 3 μL of each sample and gently mixed. A quick spin was used to concentrate the entire reaction mixture to the bottom of the tube. The reaction mixtures were incubated at 30 °C with shaking at 80 rpm for 90 min. Each reaction mixture was then aliquoted into triplicate wells containing 5 μL of the translation reaction in a 96-well black plate. Translational efficiency was measured by the addition of 100 μL of the Bright-Glo Luciferase Assay System and luminescence measured as counts per second (cps) was read on a Victor 3 (Perkin-Elmer, Shelton, CT, USA). The negative control (full translation efficiency) consisted of the translational lysate mixture with 1× PBS with 0.2% phosphate buffer gelatin. Toxin samples were diluted with 1× PBS with phosphate buffer gelatin to a final concentration of 0.2% before any experiments. Toxin activity was calculated as percentage (%) translation inhibition due to inhibition of translation [(negative control cps − toxin sample cps)/negative control cps] × 100. All values shown represent the mean ± standard deviation (SD) of triplicate samples measured in a representative experiment. Standard curves with serial dilutions of toxin were always performed in parallel to experimental conditions for each independent experiment to account for potential variations due to changes in toxin activity and other conditions. Statistical significance was calculated using the two-tailed unpaired Student’s *t*-test from GraphPad Prism 6 with *p*-values < 0.05 considered significant.

### 4.6. Vero Cell Cytotoxicity Assay

Vero cells (African green monkey kidney epithelial cells) were cultured in Dulbeco’s Modified Eagle Medium high glucose + 10% fetal bovine serum and incubated in a humidified incubator (37 °C, 5% CO_2_). Cells were trypsinized and adjusted to 5 × 10^4^ cells/mL, seeded into black-sided, clear-bottom 96-well tissue culture plates at 100 μL/well and allowed to adhere overnight (18 h) at 37 °C. The media was removed and 100 μL of fresh DMEM—containing DMEM, untreated abrin, or the toxins inactivated at different temperatures—was then added. Plates were then incubated at 37 °C for 2 h, the media was removed and more fresh media was added. The cells were then incubated for 48 h at 37 °C. Luminescence was measured as follows: 100 μL of CellTiter-Glo (Promega, G7570) at 1:5 in PBS was added to each well and the plates shaken for 2 min in order to lyse the cells. Upon another 10-min incubation at room temperature, luminescence was measured using a Victor 3 plate reader (lid was removed from the plate for a better signal). The percentage (%) cytotoxicity for each well of the toxin treated samples was calculated as follows: [(average DMEM negative control cps − experimental cps)/average DMEM negative control cps] × 100. The average % cytotoxicity was calculated for all the conditions. The non-treated or pH 7.0-treated abrin was set as 100% relative cytotoxicity. To obtain the relative cytotoxicity percentages (%) for all the other conditions, we calculated [average % cytotoxicity condition/average % cytotoxicity of positive control non-treated abrin or pH 7.0 abrin] × 100.

### 4.7. Abrin Intravenous Route (iv) Mouse Bioassay

Randomly assigned female Swiss-Webster mice in groups of two to four mice were challenged intravenously (iv) with abrin toxin resuspended in 1× PBS with 0.2% phosphate buffer gelatin per dosage level. Seven dosage levels were tested per experiment and two independent experiments were performed. Previous work from our laboratory did not find any differences in the murine strain’s susceptibility to ricin. Additionally, previous studies have used this outbred strain for both ricin and abrin [37,38]. Between the two independent experiments, *n* = 6–8 mice were tested per dosage level. Animals were monitored for at least 8 days for signs of intoxication or death. Abrin iv LD_50_ values were calculated using both the Weil method and the Reed and Muench method [33,34]. All procedures involving animals were reviewed and approved by the Institutional Animal Care and Use Committee of the United States Department of Agriculture, Western Regional Research Center. Animal use protocols for abrin mouse bioassays (Protocol # 16-2) were approved by the Western Regional Research Center Institutional Animal Care and Use Committee (WRRC- IACUC) on 2 September 2016.

## Figures and Tables

**Table 1:** In vitro Vero cell cytotoxicity assay of temperature-treated toxins.

Treatment	Relative Cytotoxicity (%)
DMEM	0
Abrin	100
99 °C	0
85 °C	0
80 °C	0
74 °C	0
63 °C	72 ± 4

Vero cell cytotoxicity after treatment with abrin, either treated or not with increasing temperatures. Abrin was used at 5 ng/mL for these assays. Values represent means of six samples ± SD. Statistical significance was determined by two-tailed unpaired Student’s *t*-test, *p* < 0.0001 for all conditions compared with abrin non-treated. Two independent experiments were performed and one representative data set is presented.

## Supplementary Materials

The following are available online at www.mdpi.com/2072-6651/9/10/320/s1, Table S1: The cytotoxic effect on Vero cells by pH-treated abrin, Figure S1: SDS-PAGE analysis of temperature (**A**) and pH-treated (**B**) toxin complexes and subunits.
