# Supplementary material for: Abrin Toxicity and Bioavailability after Temperature and pH Treatment
Source: Toxins (Basel). 2017 Oct 13;9(10):320. doi: 10.3390/toxins9100320 (PMC5666367; doi:10.3390/toxins9100320)
Supplement: Supplementary file 1 [file toxins-09-00320-s001.pdf]

Supplementary Materials: Abrin Toxicity and Bioavailability after Temperature and pH Treatment

Christina C. Tam, Thomas D. Henderson II, Larry H. Stanker, Xiaohua He and Luisa W. Cheng

Table S1: The cytotoxic effect on Vero cells by pH-treated abrin.

Treatment	Relative cytotoxicity
DMEM	0
pH 2	86 ± 2
pH 3	92 ± 1
pH 4	91 ± 1
pH 5	91 ± 1
pH 6	99 ± 0
pH 7	100
pH 8	97 ± 1
pH 9	96 ± 1

Vero cell cytotoxicity after treatment with abrin either treated or not with increasing pH. 5 ng/mL of toxin was used for this experiment. Values represent means of six samples ± SD. Statistical significance was determined by two-tailed unpaired Student's *t*-test, $p < 0.0001$ for all conditions compared with abrin at pH 7 except for pH 6 ($p = 0.2844$) and pH 8 ($p = 0.00188$). Two independent experiments were performed and one representative data set is presented.

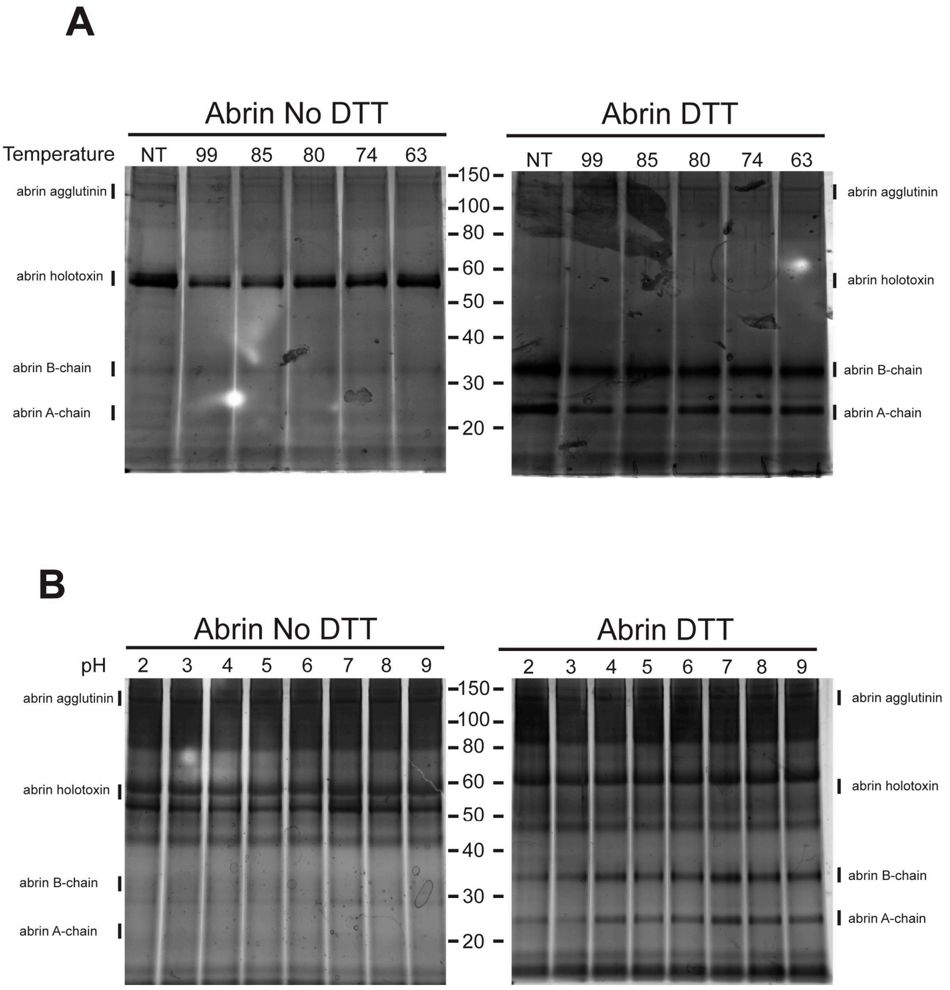

Figure S1. SDS-PAGE analysis of temperature (**A**) and pH-treated (**B**) toxin complexes and subunits. 100 ng per lane of sample (abrin not treated, temperature exposed, or pH inactivated) treated or not treated with 0.05 M DTT was loaded onto a NuPAGE 4-12% Bis-Tris gel, subjected to SDS-PAGE electrophoresis and followed by silver staining with the SilverXpress kit. The amount of abrin holotoxin and agglutinin (in No DTT samples) appears to decrease with high temperature treatment. The amount of abrin A or B-chain itself maybe affected by decreasing pH (in Abrin DTT gel).
